# Refining Outcomes in Technically Resectable Colorectal Liver Metastases: A Simplified Risk Model and the Role of Preoperative Chemotherapy

**DOI:** 10.3390/cancers18020227

**Published:** 2026-01-12

**Authors:** Kou Kanesada, Masao Nakajima, Tatsuya Ioka, Shinobu Tomochika, Yoshitaro Shindo, Yukio Tokumitsu, Hiroto Matsui, Hironori Tanaka, Yuki Nakagami, Ryouichi Tsunedomi, Michihisa Iida, Hidenori Takahashi, Hiroaki Nagano

**Affiliations:** 1 Department of Surgery, Ube Central Hospital, Ube 755-0151, Yamaguchi, Japan; 2 Department of Gastroenterological, Breast and Endocrine Surgery, Graduate School of Medicine, Yamaguchi University, Ube 755-8505, Yamaguchi, Japannakagami-yu@shimonoseki-cu.ac.jp (Y.N.); tsune-r@yamaguchi-u.ac.jp (R.T.);; 3 Oncology Center, Yamaguchi University Hospital, Ube 755-8505, Yamaguchi, Japan; 4 Health Data Science Laboratory, Faculty of Data Science, Shimonoseki City University, Shimonoseki 751-8510, Yamaguchi, Japan

**Keywords:** colorectal cancer, liver metastasis, liver resection, prognostic risk factors, preoperative chemotherapy

## Abstract

**Background**: Preoperative chemotherapy is increasingly used for colorectal liver metastases (CRLM), but simple risk stratification tools for routine practice remain limited. We developed a simple risk model to predict outcomes after curative-intent CRLM resection, including in patients receiving preoperative chemotherapy. **Methods**: We retrospectively analyzed 115 patients who underwent initial curative-intent liver resection for CRLM at two centers. Factors associated with recurrence-free survival (RFS) and overall survival (OS) were evaluated using Cox proportional hazards models and log-rank tests. Model performance was benchmarked against the Beppu nomogram and Fong’s clinical risk score using the area under the curve (AUC). Outcomes were also assessed based on response to preoperative chemotherapy. **Results**: Having ≥3 CRLMs was the only independent predictor common to both OS and RFS. Among patients with 1–2 CRLMs, the largest tumor diameter being ≥5 cm independently predicted RFS. A composite high-risk definition (≥3 CRLMs, or 1–2 CRLMs with a diameter ≥ 5 cm) independently predicted recurrence (HR 2.05, *p* = 0.007) and overall mortality (HR 2.24, *p* = 0.017). The AUCs were similar to the Beppu nomogram for recurrence (0.68 vs. 0.70 (*p* = 0.683) at 36 months, 0.66 vs. 0.68 (*p* = 0.766) at 60 months) and to Fong’s score for survival (0.59 vs. 0.64 (*p* = 0.430) at 36 months, 0.65 vs. 0.74 (*p* = 0.074) at 60 months). Among patients receiving preoperative chemotherapy (*n* = 72), high-risk status was associated with poorer RFS (HR 3.11, *p* < 0.001) and OS (HR 2.80, *p* = 0.010). Within this subgroup, progressive disease (PD) was associated with worse outcomes than disease control (CR/PR/SD). **Conclusions**: This two-variable, rule-based model provides an easy-to-use tool for postoperative risk stratification after CRLM resection, and incorporating chemotherapy response may further refine prognostication.

## Simple Summary

Preoperative chemotherapy is often given before liver resection for colorectal liver metastases, yet practical postoperative risk tools remain scarce. This retrospective study of 115 patients developed an easy, rule-based risk model using only two tumor features: the number of liver metastases and the size of the largest tumor. Patients were considered high risk if they had three or more metastases, or if they had one to two metastases with the largest tumor being 5 cm or greater. This high-risk group experienced earlier recurrence and shorter survival, and the model performed similarly to commonly used prediction tools. By focusing on two tumor features, this tool may help clinicians rapidly identify high-risk patients, guide additional therapies, and refine follow-up plans. These findings could support further research aimed at developing more personalized strategies for caring for patients with liver metastasis.

## 1. Introduction

Colorectal cancer is a leading cause of cancer-related deaths, with over one million new cases diagnosed worldwide each year [1,2]. Liver metastases are the most common cause of mortality in colorectal cancer, accounting for approximately two-thirds of all colorectal cancer deaths [3]. Recent studies have shown that new regimens combining chemotherapy (oxaliplatin or irinotecan-based) with molecular targeted agents improve progression-free survival and overall survival in metastatic colorectal cancer patients [4,5,6]. While liver resection is an effective treatment for colorectal cancer liver metastasis (CRLM), issues such as early recurrence and poor prognosis post-surgery remain prevalent [7,8]. Recurrence occurs in about two-thirds of patients who undergo liver resection, with over half experiencing recurrence within two years [9].

Due to the heterogeneous nature of CRLM, identifying prognostic factors is challenging. Numerous prognostic factors have been reported after curative resection of CRLM, including lymph node metastasis of the primary tumor, tumor markers (carcinoembryonic antigen (CEA) and carbohydrate antigen 19-9 (CA19-9)), number of liver metastases, tumor size, time from primary tumor diagnosis to liver metastasis diagnosis, and presence of extrahepatic metastases [7,10,11,12,13,14,15,16,17]. The neutrophil-lymphocyte ratio (NLR), reflecting systemic immune response, has also been reported as a prognostic marker in various malignancies, including CRLM [18]. Recently, the prognostic value of RAS status as a predictor of CRLM has also been reported [19,20,21]. Despite these findings, no treatment strategy based on these prognostic factors has been established in clinical practice, likely due to the complexity and number of variables involved.

Preoperative chemotherapy for resectable CRLM is widely used to address occult metastases and control tumor progression [22,23]. However, the benefits of perioperative chemotherapy, including preoperative chemotherapy, remain controversial. For instance, Nordlinger et al. reported in the European Organization for Research and Treatment of Cancer (EORTC) trial 40983 that, while perioperative chemotherapy with FOLFOX4 prolonged recurrence-free survival, it did not improve overall survival in patients with resectable CRLM [24,25].

Although several studies have examined risk factors for patients with CRLM undergoing liver resection, the large number of variables makes these models impractical for routine clinical use. The aim of this study was to retrospectively examine patients who underwent initial liver resection for CRLM to identify prognostic risk factors that are easy to apply in clinical practice. Additionally, we evaluated the impact of perioperative chemotherapy on CRLM outcomes in relation to these prognostic risk factors.

## 2. Materials and Methods

### 2.1. Patient Data

This was a multi-center retrospective observational study. We enrolled 115 consecutive patients with CRLM who underwent initial liver resection with R0 surgery (excluding 17 patients with R1/R2 surgery) at the Department of Gastroenterological Surgery, Yamaguchi University Graduate School of Medicine, and the Department of Surgery at Ube Central Hospital, between 2010 and 2021. Ube Central Hospital is affiliated with Yamaguchi University Hospital, and institutional practices for CRLM management—including surgical indications, perioperative care, and follow-up surveillance—were aligned between the two centers, as detailed below. The decision regarding resectability was made at a conference involving hepatobiliary and pancreatic surgeons at each institution. We defined “technically resectable” for CRLMs as those with no proximity to three hepatic veins, no proximity to the left or right portal branches, and a residual liver volume of 30% or more.

Data regarding perioperative chemotherapy were obtained. The therapeutic effects of preoperative chemotherapy were evaluated according to the RECIST ver. 1.1 [26]. The radiologic response assessments, including imaging modality and timing relative to surgery, were performed at a conference involving hepatobiliary and pancreatic surgeons at each institution.

Regarding the timing of diagnosis, synchronous liver metastases were defined as those present at the time of the primary tumor diagnosis or appearing within one year after primary tumor resection. Metachronous liver metastases were defined as those appearing more than one year after primary tumor resection. The size and number of liver metastatic lesions were measured at the time of liver metastasis diagnosis using abdominal ultrasonography, computed tomography scan, and gadoxetic acid-enhanced magnetic resonance imaging (EOB-MRI). Beyond preoperative imaging, real-time navigation systems for laparoscopic hepatectomy are being developed to support intraoperative orientation and lesion targeting [27]. Hybrid tracking approaches enabling more accurate laparoscopic ultrasound reconstruction represent another promising direction to improve intraoperative spatial understanding during minimally invasive liver surgery [28]. Accordingly, real-time navigational guidance was applied to select laparoscopic hepatectomies, which helped maintain intraoperative spatial awareness and guide accurate tumor-directed resection. CT scans and EOB-MRI served as the primary modalities for preoperative staging, with ultrasound used as an adjunct when applicable. Where multiple modalities were available, we specified the hierarchy used to define tumor number and maximal diameter (e.g., CT/MRI prioritized; largest recorded diameter used). Follow-up after R0 resection involves mainly CT scans every 3 to 6 months, and we added EOB-MRI or PET-CT as needed to evaluate for liver metastasis recurrence or extrahepatic recurrence.

This study was approved by the Human Ethics Committee of Yamaguchi University (approval no. H2025-005), and all patients provided written informed consent. This study was conducted in accordance with the Declaration of Helsinki.

### 2.2. Variables Evaluated for Univariate Analysis

Fourteen perioperative predictor variables were evaluated. A univariate analysis of prognostic risk factors associated with recurrence-free survival (RFS) after R0 resection and overall survival (OS) after liver resection included age, gender, primary site, primary tumor T status, lymph node metastasis status of the primary tumor, lymphatic invasion of the primary tumor, venous invasion of the primary tumor, CEA level at diagnosis of liver metastasis, CA19-9 level at diagnosis of liver metastasis, number of CRLMs, diameter of the largest tumor, timing of diagnosis of CRLM, presence of extrahepatic metastatic disease, and NLR measured before liver resection. The cutoff values for the number of liver metastases and NLR were 3 and 2.9, respectively. The receiver operating characteristic (ROC) curve analysis for recurrence using logistic regression was used to determine the cutoff values by maximizing Youden’s index (defined as sensitivity + specificity − 1) for each variable.

### 2.3. Statistical Analysis

Univariate and multivariate analyses based on the Cox proportional hazard model were used to identify risk factors related to RFS and OS. Missing values were handled using the complete case method. All variables with a *p*-value < 0.10 in the univariate analysis were included in the multivariate analysis. Kaplan–Meier survival curves with log-rank tests were used to evaluate RFS and OS. The models were evaluated by the time-dependent ROC curve analysis using a certain time period. All statistical analyses were performed using JMP Pro 18 software (SAS Institute, Cary, NC, USA), with the exception of the time-dependent ROC curve analysis, which was performed using the statistical programming language R (version 4.4.1, R Development Core Team, Vienna, Austria). A *p*-value < 0.05 was considered statistically significant.

## 3. Results

### 3.1. Subsection Patient Background

Table 1 shows the clinical background of the 115 patients. Lymph node metastasis at the time of primary colorectal resection was present in 76 patients (66.1%). CEA levels > 5.0 ng/mL at the diagnosis of liver metastasis were observed in 85 patients (73.9%), and CA19-9 levels > 37.0 U/mL were observed in 39 patients (33.9%). The number of liver metastases at diagnosis was ≥3 in 31 patients (27.0%). The diameter of the largest tumor at diagnosis was ≥5 cm in 22 patients (19.1%). NLR was ≥2.9 in 26 patients (22.6%). Missing values were observed for primary tumor T status in one patient (0.9%), primary tumor lymph node status in three patients (2.6%), lymphatic vessel invasion in seven patients (6.1%), and venous invasion in six patients (5.2%). And also, there were RAS status, tested in *n* = 85 (73.9%), missing in *n* = 30 (26.1%); BRAF status, tested in *n* = 47 (40.9%), missing in *n* = 68 (59.1%); and Microsatellite Instability (MSI) status, tested in *n* = 21 (18.3%), missing in *n* = 94 (81.7%).

Preoperative chemotherapy was administered to 72 patients (62.6%), with 56 receiving doublet regimens (CAPOX, FOLFOX, or FOLFIRI), 7 receiving a triplet regimen (FOLFOXIRI), and 9 receiving other regimens. Molecular-targeted drugs were added in 49 patients. Among 72 patients who received preoperative chemotherapy prior to hepatectomy, 54 (75.0%) underwent neoadjuvant therapy for technically resectable CRLM, whereas 18 (25.0%) underwent conversion-intent therapy for initially technically and oncologically unresectable CRLM. Given the long inclusion period, the cohort was stratified into two treatment eras (2010–2015, *n* = 38; 2016–2021, *n* = 34) to describe secular changes in systemic therapy. Compared with 2010–2015, the later era showed a higher proportion of neoadjuvant therapy (94.1% [32/34] vs. 57.9% [22/38]) with fewer conversion cases (5.9% [2/34] vs. 42.1% [16/38]; Fisher’s exact test, *p* = 0.003). Doublet chemotherapy was less frequently used in 2016–2021 (76.5% [26/34] vs. 94.7% [36/38]; *p* = 0.039), whereas triplet regimens were introduced in the later era (20.6% [7/34] vs. 0% [0/38]; *p* = 0.004). The use of molecular-targeted agents decreased from 94.7% (36/38) to 79.4% (27/34) across eras (*p* = 0.075). Postoperative chemotherapy was administered to 26 patients (22.6%), with 14 receiving doublet regimens (CAPOX, FOLFOX, or FOLFIRI), 9 receiving UFT plus UZEL, and 3 patients receiving other regimens.

Regarding the timing of diagnosis, synchronous diagnosis was observed in 95 patients (82.6%). Extrahepatic metastases at the time of liver metastasis diagnosis were found in 14 patients (12.2%).

### 3.2. Recurrence-Free Survival and Overall Survival

Supplementary Figure S1 shows the analysis of RFS and OS of the patients after R0 resection for CRLM (*n* = 115). With a median follow-up period of 46 months (range, 6–153 months), the median RFS was 10 months, and the 5-year RFS probability was 29.9%. Median OS time was not reached, and the 5-year OS probability was 58.7%.

### 3.3. Univariate and Multivariate Analysis of Risk Factors Associated with Recurrence After R0 Resection and Survival After Liver Resection

The univariate and multivariate analyses of risk factors associated with RFS are summarized in Table 2. Univariate analysis showed that CEA level (≥5.0 ng/mL), CA19-9 level (≥37.0 U/mL), number of CRLMs (≥3), diameter of the largest tumor (≥5 cm), and NLR (≥2.9) were significantly associated with recurrence. In the multivariate analysis, number of CRLMs (≥3) (hazard ratio [HR] = 2.54, 95% confidence interval [CI]: 1.51–4.19, *p* ≤ 0.001) and NLR (≥2.9) (HR = 2.16, 95% CI: 1.20–3.76, *p* = 0.012) were identified as independent risk factors for recurrence after R0 resection.

The univariate and multivariate analyses of risk factors associated with OS are summarized in Table 3. The univariate analysis showed that CEA level (≥5.0 ng/mL) and the number of CRLMs (≥3) were significantly associated with mortality. In the multivariate analysis, CEA level (≥5.0 ng/mL) (HR = 2.41, 95% CI: 1.00–7.15, *p* = 0.049) and number of CRLMs (≥3) (HR = 2.60, 95% CI: 1.34–4.97, *p* = 0.005) were identified as independent risk factors for OS after liver resection. These results indicated that the number of CRLMs ≥ 3 may be a significant risk factor for both recurrence and survival in CRLM.

**Figure 1:**
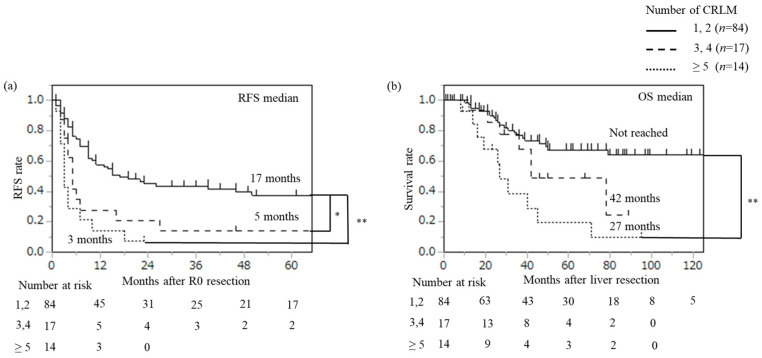
Recurrence-free survival (RFS) after R0 resection (**a**) and overall survival (OS) after liver resection (**b**) for CRLM according to number of CRLMs: 1 or 2 versus 3 or 4 versus ≥ 5. Kaplan–Meier curves between each group were analyzed by the log-rank test based on Bonferroni correction. * < 0.01, ** < 0.001 (the log-rank test).

**Figure 2:**
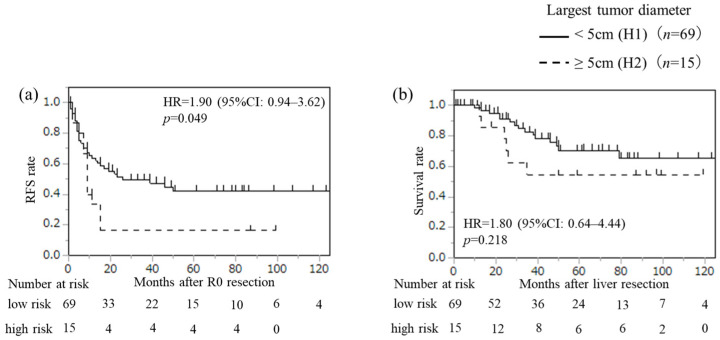
Recurrence-free survival (RFS) after R0 resection (**a**) and overall survival (OS) after liver resection (**b**) in patients with 1 or 2 CRLMs: <5 cm (H1) versus ≥ 5 cm (H2). Kaplan–Meier curves between the two groups were analyzed by the log-rank test.

We further examined the impact of the number of CRLMs on prognosis in detail. Number of CRLMs was divided into three categories: 1, 2 (*n* = 84), 3, 4 (*n* = 17), and ≥5 (*n* = 14), and recurrence and survival were examined using Kaplan–Meier curves (Figure 1). Median RFS was 17 months in patients with 1 or 2 CRLMs, 5 months in those with 3 or 4 CRLMs, and 3 months in those with ≥5 CRLMs. There was a significant difference in RFS between patients with 1 or 2 CRLMs and those with 3 or 4 CRLMs (*p* = 0.009, based on Bonferroni correction). On the other hand, there was no difference in RFS between patients with 3 or 4 CRLMs and those with ≥5 CRLMs (*p* = 0.170). Median OS time was not reached in patients with 1 or 2 CRLM. In contrast, median OS time was 42 months in patients with 3 or 4 CRLMs and 27 months in those with ≥5 CRLMs. There was a significant difference in OS between patients with 1 or 2 CRLMs and those with ≥5 CRLMs (*p* < 0.001), while there was no difference between patients with 3 or 4 CRLMs and those with ≥5 CRLMs (*p* = 0.087). In patients with 1 or 2 CRLMs, only the diameter of the largest tumor ≥ 5 cm was identified as a significant predictor for recurrence (*p* = 0.049) (Figure 2). Thus, the number of CRLMs (≥3) and the diameter of the largest tumor ≥ 5 cm in patients with 1 or 2 CRLMs could serve as novel prognostic criteria for predicting high-risk groups in patients with CRLM who underwent initial liver resection.

### 3.4. Novel Prognostic Criteria of CRLM After Curative Resection

**Figure 3:**
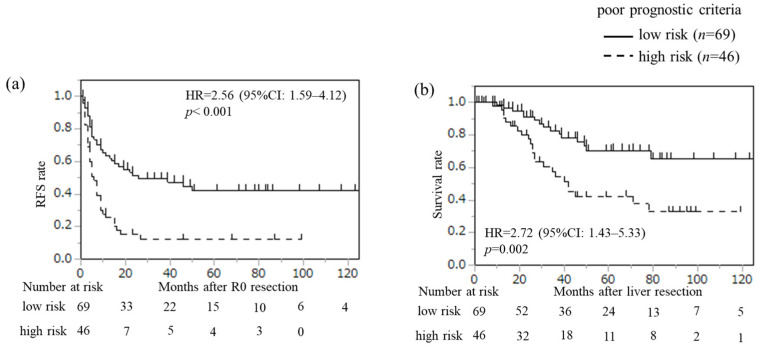
Recurrence-free survival (RFS) after R0 resection (**a**) and overall survival (OS) after liver resection (**b**) for CRLM based on poor prognostic criteria (*n* = 115): low-risk group versus high-risk group. Kaplan–Meier curves between the two groups were analyzed by the log-rank test.

**Figure 4:**
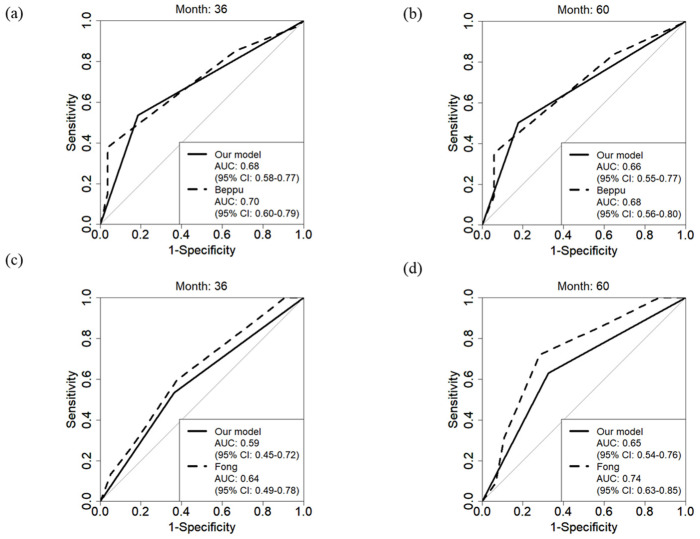
Time-dependent receiver operating characteristic (ROC) curve analysis on recurrence-free survival (RFS) (**a**,**b**) and overall survival (OS) (**c**,**d**).

“The number of CRLMs (≥3)” or “diameter of the largest tumor ≥ 5 cm in patients with 1 or 2 CRLMs” are each defined as risk factors, and cases exhibiting either factor are classified as the high-risk group. Figure 3 shows the Kaplan–Meier curves of RFS and OS according to the novel prognostic criteria. The high-risk group and elevated NLR, as defined by these prognostic criteria, were significantly associated with increased risk of recurrence (Table S1; HR = 2.05, 95% CI: 1.22–3.45, *p* = 0.007, and HR = 2.00, 95% CI: 1.11–3.47, *p* = 0.022, respectively) and reduced survival (Table S2; HR = 2.34, 95% CI: 1.16–4.47, *p* = 0.017). We calculated the area under the curve (AUC) to assess the predictive accuracy of our model for recurrence and OS, applying time-dependent ROC curve analysis at 36- and 60-month points (Figure 4). The AUCs for recurrence in our model were 0.68 (95% CI: 0.58–0.77) at 36 months and 0.66 (95% CI: 0.55–0.77) at 60 months. For OS, AUCs were 0.59 (95% CI: 0.45–0.72) at 36 months and 0.65 (95% CI: 0.54–0.76) at 60 months. For comparison, the Beppu nomogram yielded AUCs of 0.70 (*p* = 0.683) at 36 months and 0.68 (*p* = 0.766) at 60 months for recurrence, and Fong’s clinical risk score resulted in AUCs of 0.64 (*p* = 0.430) at 36 months and 0.74 (*p* = 0.074) at 60 months for OS. When this risk model was examined in the preoperative chemotherapy group (*n* = 72), there was a significant difference in RFS (HR = 3.11, 95% CI: 1.68–6.04, *p* < 0.001) and OS (HR = 2.80, 95% CI: 1.28–6.76, *p* = 0.010) between the low-risk and high-risk groups.

### 3.5. RFS and OS Related to Therapeutic Effects of Preoperative Chemotherapy

**Figure 5:**
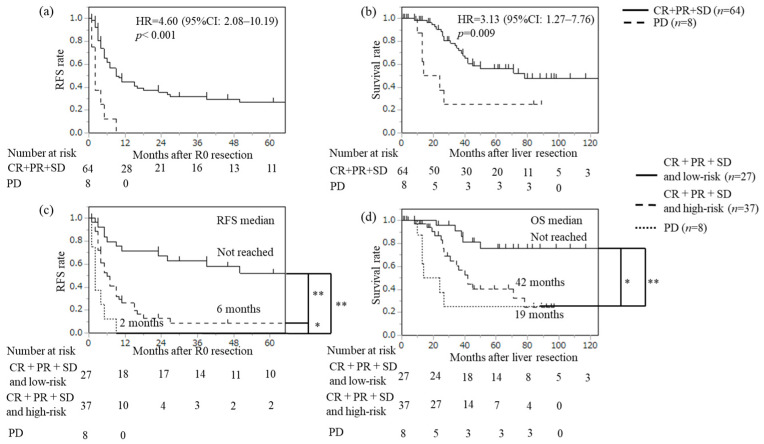
Recurrence-free survival (RFS) (**a**,**c**) and overall survival (OS) (**b**,**d**) after R0 resection for CRLM according to therapeutic effects of preoperative chemotherapy (*n* = 72): CR + PR + SD versus PD. (**c**,**d**) CR + PR + SD and low-risk group, CR + PR + SD and high-risk group vs. PD. Kaplan–Meier curves between the two groups were analyzed by the log-rank test. * < 0.01, ** < 0.001 (the log-rank test based on Bonferroni correction).

The numbers of patients with complete response (CR), partial response (PR), stable disease (SD), and progressive disease (PD) were 1, 32, 31, and 8, respectively. Both RFS and OS were significantly worse in the PD group than in the CR + PR + SD group (RFS: HR = 4.60, 95% CI: 2.08–10.19, *p* < 0.001; OS: HR = 3.13, 95% CI: 1.27–7.76, *p* = 0.009) (Figure 5a,b). Additionally, when the CR + PR + SD group was divided into low-risk and high-risk groups using these prognostic criteria, there was a significant difference in RFS between the low-risk and high-risk groups (*p* < 0.001). And also, OS showed a significantly poorer prognosis in the high-risk group (*p* = 0.002) (Figure 5c,d).

## 4. Discussion

In this study, the number of CRLMs (≥3) was identified as a risk factor for recurrence and survival after initial liver resection. Furthermore, among patients with only 1 or 2 CRLMs, tumor diameter ≥ 5 cm was associated with a high risk of recurrence. Previous studies have established tumor diameter ≥ 5 cm as a significant prognostic criterion [7,12], and similarly, the number of CRLMs ≥ 5 has been associated with poor prognosis [12]. In the present study, the proportions of patients with 3 or 4 CRLMs and those with ≥5 CRLMs at risk of recurrence were similar (Figure 1). Regarding prognosis, these groups also had similar proportions of those with long-term survival. Thus, patients with ≥3 CRLMs might represent a group with poor prognosis and may face challenges in achieving a complete cure.

Fong et al. [7] reported a clinical risk score for OS in patients with CRLM using five clinical criteria: nodal status in primary cancer, disease-free interval from the primary to discovery of the liver metastases, number of tumors, preoperative CEA level, and size of the largest tumor. Beppu et al. [12] also reported on a Japanese Society of Hepato-Biliary-Pancreatic Surgery nomogram for predicting RFS in patients with CRLM undergoing upfront hepatectomy, combining six risk factors: timing of liver metastasis diagnosis, presence of lymph node metastasis, number of liver metastases, maximum tumor size of liver metastasis, presence of extrahepatic metastasis, and tumor marker (CA19-9). While these models are robust, they often require a complex set of variables, which can hinder routine use in clinical practice. Our model, in contrast, employs only two key factors—number of CRLMs and tumor diameter—to predict prognosis, aiming for simplicity without sacrificing accuracy. The AUC applying time-dependent ROC curve analysis obtained in this study indicate that our model’s predictive accuracy is comparable to that of the Beppu nomogram for recurrence (0.68 vs. 0.70 (*p* = 0.683) at 36 months and 0.66 vs. 0.68 (*p* = 0.766) at 60 months) and Fong’s score for OS (0.59 vs. 0.64 (*p* = 0.430) at 36 months and 0.65 vs. 0.74 (0.074) at 60 months). This suggests that our model could offer a more practical tool for routine clinical use while maintaining a level of accuracy similar to more complex scoring systems. And also, we emphasize practical scenarios where a two-factor tool may be advantageous in rapid counseling, triage for neoadjuvant consideration, and communication in multidisciplinary settings.

In this study, high NLR (cutoff value: 2.9) was identified as a risk factor in recurrence after R0 resection. NLR has been reported to be a prognostically relevant marker for various gastrointestinal cancers, including CRLM [18]. Elevated NLR has been linked to poorer survival outcomes in CRLM patients, likely due to the inflammatory response promoting angiogenesis and suppressing immune surveillance, which facilitates tumor progression [29]. However, the optimal cutoff value of NLR remains undetermined and warrants future investigation.

Preoperative chemotherapy is sometimes administered in the treatment of CRLM in daily clinical practice. When the risk model in this study was examined in the preoperative chemotherapy group, there was a significant difference in RFS and OS between the low-risk and the high-risk groups. Therefore, we believe that our novel risk model may also be effective for predicting recurrence in patients receiving preoperative chemotherapy.

While preoperative chemotherapy has been reported to be associated with a reduction in recurrence, its direct impact on survival remains unproven [24,25]. In EORTC trial 40983, the potential factor contributing to the limited benefit of preoperative chemotherapy could be the inclusion of mainly low-risk patients, excluding patients with ≥five CRLM [25]. In contrast, a study by Ichida et al. [30] revealed positive outcomes with preoperative chemotherapy for resectable CRLM using strict criteria. Notably, administering preoperative chemotherapy to high-risk patients with at least four CRLMs, a tumor diameter ≥ 5 cm, and a resectable extrahepatic metastatic disease resulted in improved survival [30]. Tailoring preoperative chemotherapy for high-risk profiles may be associated with enhanced overall survival after curative resection for resectable CRLM.

The clinical utility of our model is enhanced by its potential applications in decision-making processes for perioperative chemotherapy. For instance, identifying high-risk patients based on a CRLM count of ≥3 and tumor diameter could support the selection of intensive chemotherapy regimens, such as FOLFOXIRI, in patients who are more likely to benefit from aggressive treatment approaches. This stratification could further guide follow-up strategies, allowing for tailored monitoring schedules based on individual risk profiles. And also, Additionally, the simplicity of our model allows for broader applicability across various healthcare settings, including those with limited resources. By reducing the number of variables needed to assess risk, this model can be more easily implemented internationally, potentially serving as a practical guideline for CRLM management beyond highly specialized centers.

Administering stronger chemotherapy drugs like FOLFOXIRI to these patients could potentially improve prognosis [31,32,33]. However, caution should be taken regarding severe adverse events, such as neutropenia and liver dysfunction after hepatectomy, when administering potent chemotherapy drugs preoperatively [34,35]. We plan to conduct a prospective study to evaluate the efficacy of FOLFOXIRI as a neoadjuvant chemotherapy drug for high-risk patients with resectable CRLM using this prognostic criterion.

Nonetheless, this study has several limitations. Its retrospective design, historical (treatment-era) bias, and modest sample size may have limited precision and left residual confounding. Because missing data necessitated a complete-case analysis, selection bias may have been introduced, and the generalizability of the findings may be reduced. Systemic therapy was not fully standardized: indications for chemotherapy and regimens were heterogeneous, and during the later study period (2016–2021), seven patients received a triplet regimen. As current evidence has not established a clear survival advantage of neoadjuvant triplet therapy over standard doublet regimens in CRLM, any influence of this small subgroup on survival estimates is likely limited; nevertheless, the role of triplet therapy in this setting warrants further study. In addition, the study included only two institutions; however, both are affiliated with Yamaguchi University, which may have mitigated inter-site variation in surgical and medical management as well as RECIST-based response assessment. Finally, imaging was interpreted locally without central radiology review, and metastatic burden was abstracted primarily as lesion number and size. This approach precluded reliable assessment of bilobar distribution and may have introduced measurement variability across modalities and centers, potentially attenuating associations and underestimating model performance. Moreover, several prognostic variables increasingly emphasized in the contemporary literature—such as margin width and molecular characteristics (RAS/BRAF mutation and MSI status)—were unavailable, and the inability to adjust for these factors may represent an additional source of bias.

Future studies, particularly multicenter prospective trials with prespecified treatment approaches, are necessary to validate these findings and further refine this model’s utility in guiding clinical decisions for CRLM patients. More broadly, the growing minimally invasive liver surgery literature—including recent meta-analytic evidence—suggests that laparoscopic liver resection can reduce length of stay and morbidity without clear compromise in oncologic surrogates in selected settings [36]. Because postoperative complications—particularly in older patients—are associated with worse oncologic outcomes after cancer surgery, future studies should evaluate surgical approach and technique as prognostic variables and consider incorporating them into novel postoperative prognostic criteria after CRLM resection.

## 5. Conclusions

In conclusion, the presence of CRLM ≥ 3 or the largest tumor diameter being ≥ 5 cm among patients with 1 or 2 CRLM may serve as a novel prognostic criterion for identifying high-risk patients following initial liver resection for CRLM. By focusing on only two tumor features, this tool may help clinicians rapidly identify high-risk patients, guide additional therapies, and refine follow-up plans. These findings may support further research toward more personalized strategies for CRLM patients.

## Figures and Tables

**Table 1:** Patient characteristics of CRLM (*n* = 115).

	*n* = 115	%
Age (years-old) *	68 (34–84)	
Gender		
Male	71	61.7
Female	44	38.3
Primary site		
Right	38	33.0
Left	77	67.0
Primary tumor T status		
pT1-3	91	79.1
pT4	23	20.0
Unknown	1	0.9
Primary tumor LN status		
Negative	36	31.3
Positive	76	66.1
Unknown	3	2.6
ly (primary tumor)		
Negative	13	11.3
Positive	95	82.6
Unknown	7	6.1
v (primary tumor)		
Negative	27	23.5
Positive	82	71.3
Unknown	6	5.2
CEA level **		
≤5.0 ng/mL	30	26.1
>5.0 ng/mL	85	73.9
CA19-9 level **		
≤37.0 U/mL	76	66.1
>37.0 U/mL	39	33.9
Number of CRLM **		
1, 2	84	73.0
3, 4	17	14.8
≥5	14	12.2
Largest tumor diameter **		
<5 cm	93	80.9
≥5 cm	22	19.1
Timing of diagnosis of CRLM ***		
Synchronous	95	82.6
Metachronous	20	17.4
Extrahepatic metastatic disease **		
Yes	14	12.2
No	101	87.8
NLR ratio ****		
<2.9	89	77.4
≥2.9	26	22.6
Preoperative chemotherapy		
Yes	72	62.6
No	43	37.4
Postoperative chemotherapy		
Yes	26	22.6
No	89	77.4
RAS status		
Tested	85	73.9
Missing	30	26.1
BRAF status		
Tested	47	40.9
Missing	68	59.1
MSI status		
Tested	21	18.3
Missing	94	81.7

* Median (range), ** at diagnosis of CRLM, *** Synchronous: Liver metastases are present at the time of diagnosis of the primary tumor, or liver metastases appear within 1 year after resection of the primary tumor. Metachronous: Liver metastases appear more than 1 year after resection of the primary tumor, **** at before liver resection. ly: lymphatic vascular invasion, v: venous invasion, CEA: carcinoembryonic antigen, CA19-9: carbohydrate antigen 19-9, NLR: neutrophil–lymphocyte ratio, MSI: Microsatellite Instability.

**Table 2:** Univariate and multivariate analysis of risk factors associated with recurrence after R0 resection for CRLM (*n* = 115).

Risk Factors		*n* (%)	Univariate				Multivariate			
			HR	95%CI		*p* Value	HR	95%CI		*p* Value
Age (years-old)	<65	44 (38.3)	1	-	-	-				
	≥65	71 (61.7)	1.10	0.69	1.78	0.701				
Gender	Male	71 (61.7)	1	-	-	-				
	Female	44 (38.3)	1.07	0.66	1.71	0.768				
Primary site	Right	38 (33.0)	1	-	-	-				
	Left	77 (67.0)	0.90	0.56	1.48	0.679				
Primary tumor T status	pT1-3	91 (79.1)	1	-	-	-				
	pT4	23 (20.0)	0.98	0.55	1.68	0.954				
Primary tumor LN status	Negative	36 (31.3)	1	-	-	-				
	Positive	76 (66.1)	1.28	0.77	2.19	0.348				
ly (primary tumor)	Negative	13 (11.3)	1	-	-	-	1	-	-	-
	Positive	95 (82.6)	1.92	0.90	4.98	0.096	1.69	0.78	4.45	0.198
v (primary tumor)	Negative	27 (23.5)	1	-	-	-				
	Positive	82 (71.3)	0.89	0.53	1.59	0.683				
CEA level *	≤5.0 ng/mL	30 (26.1)	1	-	-	-	1	-	-	-
	>5.0 ng/mL	85 (73.9)	1.83	1.06	3.36	0.029	1.31	0.71	2.56	0.396
CA19-9 level *	≤37.0 U/mL	76 (66.1)	1	-	-	-	1	-	-	-
	>37.0 U/mL	39 (33.9)	1.66	1.03	2.66	0.039	1.13	0.66	1.93	0.654
Number of CRLM *	1, 2	84 (73.0)	1	-	-	-	1	-	-	-
	≥3	31 (27.0)	2.69	1.63	4.37	<0.001	2.54	1.51	4.19	<0.001
Largest tumor diameter *	<5 cm	93 (80.9)	1	-	-	-	1	-	-	-
	≥5 cm	22 (19.1)	1.80	1.03	3.01	0.039	1.26	0.69	2.24	0.439
Timing of diagnosis of CRLM **	Metachronous	20 (17.4)	1	-	-	-				
	Synchronous	95 (82.6)	1.67	0.90	4.37	0.110				
Extrahepatic metastatic disease *	No	101 (87.8)	1	-	-	-				
	Yes	14 (12.2)	1.29	0.62	2.40	0.472				
NLR ***	<2.9	89 (77.4)	1	-	-	-	1	-	-	-
	≥2.9	26 (22.6)	2.18	1.26	3.62	0.006	2.16	1.20	3.76	0.012

Univariate and multivariate analyses were performed using the Cox proportional hazards model. In the multivariate analysis, factors were extracted based on *p* values less than 0.10. * at diagnosis of CRLM. ** Synchronous: Liver metastases are present at the time of diagnosis of the primary tumor, or liver metastases appear within 1 year after resection of the primary tumor. Metachronous: Liver metastases appear more than 1 year after resection of the primary tumor, *** before liver resection. ly: lymphatic vascular invasion, v: venous invasion, CEA: carcinoembryonic antigen, CA19-9: carbohydrate antigen 19-9, NLR: neutrophil–lymphocyte ratio.

**Table 3:** Univariate and multivariate analysis of risk factors associated with survival after liver resection for CRLM (*n* = 115).

Risk Factors		*n* (%)	Univariate				Multivariate			
			HR	95%CI		*p* Value	HR	95%CI		*p* Value
Age (years-old)	<65	44 (38.3)	1	-	-	-				
	≥65	71 (61.7)	1.59	0.83	3.22	0.166				
Gender	Male	71 (61.7)	1	-	-	-				
	Female	44 (38.3)	1.25	0.65	2.38	0.494				
Primary site	Right	38 (33.0)	1	-	-	-				
	Left	77 (67.0)	1.46	0.73	3.15	0.294				
Primary tumor T status	pT1-3	91 (79.1)	1	-	-	-				
	pT4	23 (20.0)	0.60	0.23	1.35	0.232				
Primary tumor LN status	Negative	36 (31.3)	1	-	-	-				
	Positive	76 (66.1)	1.73	0.83	4.06	0.147				
ly (primary tumor)	Negative	13 (11.3)	1	-	-	-				
	Positive	95 (82.6)	1.37	0.55	4.59	0.535				
v (primary tumor)	Negative	27 (23.5)	1	-	-	-				
	Positive	82 (71.3)	0.73	0.37	1.54	0.397				
CEA level *	≤5.0 ng/mL	30 (26.1)	1	-	-	-	1	-	-	-
	>5.0 ng/mL	85 (73.9)	2.98	1.27	8.72	0.010	2.41	1.00	7.15	0.049
CA19-9 level *	≤37.0 U/mL	76 (66.1)	1	-	-					
	>37.0 U/mL	39 (33.9)	1.18	0.60	2.24	0.622				
Number of CRLM *	1, 2	84 (73.0)	1	-	-	-	1	-	-	-
	≥3	31 (27.0)	2.92	1.51	5.57	0.002	2.60	1.34	4.97	0.005
Largest tumor diameter *	<5 cm	93 (80.9)	1	-	-	-				
	≥5 cm	22 (19.1)	1.76	0.83	3.45	0.132				
Timing of diagnosis of CRLM **	Metachronous	20 (17.4)	1	-	-	-				
	Synchronous	95 (82.6)	1.40	0.60	4.10	0.462				
Extrahepatic metastatic disease *	No	101 (87.8)	1	-	-	-				
	Yes	14 (12.2)	1.14	0.39	2.68	0.786				
NLR ***	<2.9	89 (77.4)	1	-	-	-	1	-	-	-
	≥2.9	26 (22.6)	2.23	0.94	4.71	0.068	1.70	0.71	3.65	0.216

Univariate and multivariate analyses were performed using the Cox proportional hazards model. In the multivariate analysis, factors were extracted based on *p* values less than 0.10. * at diagnosis of CRLM. ** Synchronous: Liver metastases are present at the time of diagnosis of the primary tumor, or liver metastases appear within 1 year after resection of the primary tumor. Metachronous: Liver metastases appear more than 1 year after resection of the primary tumor, *** before liver resection. ly: lymphatic vascular invasion, v: venous invasion, CEA: carcinoembryonic antigen, CA19-9: carbohydrate antigen 19-9, NLR: neutrophil–lymphocyte ratio.

## Data Availability

The original contributions presented in this study are included in the article/Supplementary Material. Further inquiries can be directed to the corresponding author.

## Supplementary Materials

The following supporting information can be downloaded at: https://www.mdpi.com/article/10.3390/cancers18020227/s1, Figure S1. Analysis of RFS after R0 resection and OS after liver resection of the patients with CRLM (*n* = 115). Table S1. Univariate and multivariate analyses of risk factors associated with recurrence after R0 resection for CRLM (*n* = 115). Table S2. Univariate and multivariate analysis of risk factors associated with survival after liver resection for CRLM (*n* = 115).

## Abbreviations

The following abbreviations are used in this manuscript:

AUC	Area under the curve
CRLM	Colorectal liver metastasis
CEA	Carcinoembryonic antigen
CA19-9	Carbohydrate antigen 19-9
CI	Confidence interval
CR	Complete response
EORTC	European Organization for Research and Treatment of Cancer
HR	Hazard ratio
MSI	Microsatellite Instability
NLR	Neutrophil–lymphocyte ratio
RFS	Recurrence-free survival
OS	Overall survival
PD	Progressive disease
PR	Partial response
ROC	Receiver operating characteristic
SD	Stable disease

## References

[1] Siegel R.L., Miller K.D., Fuchs H.E., Jemal A. (2021). Cancer Statistics, 2021. CA Cancer J. Clin..

[2] Arnold M., Sierra M.S., Laversanne M., Soerjomataram I., Jemal A., Bray F. (2017). Global patterns and trends in colorectal cancer incidence and mortality. Gut.

[3] Zarour L.R., Anand S., Billingsley K.G., Bisson W.H., Cercek A., Clarke M.F., Coussens L.M., Gast C.E., Geltzeiler C.B., Hansen L. (2017). Colorectal Cancer Liver Metastasis: Evolving Paradigms and Future Directions. Cell. Mol. Gastroenterol. Hepatol..

[4] Saltz L.B., Clarke S., Díaz-Rubio E., Scheithauer W., Figer A., Wong R., Koski S., Lichinitser M., Yang T.S., Rivera F. (2008). Bevacizumab in combination with oxaliplatin-based chemotherapy as first-line therapy in metastatic colorectal cancer: A randomized phase III study. J. Clin. Oncol..

[5] Saltz L.B., Cox J.V., Blanke C., Rosen L.S., Fehrenbacher L., Moore M.J., Maroun J.A., Ackland S.P., Locker P.K., Pirotta N. (2000). Irinotecan plus fluorouracil and leucovorin for metastatic colorectal cancer. Irinotecan Study Group. N. Engl. J. Med..

[6] Douillard J.Y., Siena S., Cassidy J., Tabernero J., Burkes R., Barugel M., Humblet Y., Bodoky G., Cunningham D., Jassem J. (2010). Randomized, phase III trial of panitumumab with infusional fluorouracil, leucovorin, and oxaliplatin (FOLFOX4) versus FOLFOX4 alone as first-line treatment in patients with previously untreated metastatic colorectal cancer: The PRIME study. J. Clin. Oncol..

[7] Fong Y., Fortner J., Sun R.L., Brennan M.F., Blumgart L.H. (1999). Clinical score for predicting recurrence after hepatic resection for metastatic colorectal cancer: Analysis of 1001 consecutive cases. Ann. Surg..

[8] Adam R., Pascal G., Azoulay D., Tanaka K., Castaing D., Bismuth H. (2003). Liver resection for colorectal metastases: The third hepatectomy. Ann. Surg..

[9] de Jong M.C., Pulitano C., Ribero D., Strub J., Mentha G., Schulick R.D., Choti M.A., Aldrighetti L., Capussotti L., Pawlik T.M. (2009). Rates and patterns of recurrence following curative intent surgery for colorectal liver metastasis: An international multi-institutional analysis of 1669 patients. Ann. Surg..

[10] Rees M., Tekkis P.P., Welsh F.K., O’Rourke T., John T.G. (2008). Evaluation of long-term survival after hepatic resection for metastatic colorectal cancer: A multifactorial model of 929 patients. Ann. Surg..

[11] Kanemitsu Y., Kato T. (2008). Prognostic models for predicting death after hepatectomy in individuals with hepatic metastases from colorectal cancer. World J. Surg..

[12] Beppu T., Sakamoto Y., Hasegawa K., Honda G., Tanaka K., Kotera Y., Nitta H., Yoshidome H., Hatano E., Ueno M. (2012). A nomogram predicting disease-free survival in patients with colorectal liver metastases treated with hepatic resection: Multicenter data collection as a Project Study for Hepatic Surgery of the Japanese Society of Hepato-Biliary-Pancreatic Surgery. J. Hepato-Biliary Pancreat. Sci..

[13] Saiura A., Yamamoto J., Hasegawa K., Koga R., Sakamoto Y., Hata S., Makuuchi M., Kokkudo N. (2012). Liver resection for multiple colorectal liver metastases with surgery up-front approach: Bi-institutional analysis of 736 consecutive cases. World J. Surg..

[14] Hokuto D., Nomi T., Yamato I., Yasuda S., Obara S., Yoshikawa T., Kawaguchi C., Yamada T., Kanehiro H., Nakajima Y. (2016). The prognosis of liver resection for patients with four or more colorectal liver metastases has not improved in the era of modern chemotherapy. J. Surg. Oncol..

[15] Beppu T., Yamamura K., Sakamoto K., Honda G., Kobayashi S., Endo I., Hasegawa K., Kotake K., Itabashi M., Hashiguchi Y. (2023). Validation study of the JSHBPS nomogram for patients with colorectal liver metastases who underwent hepatic resection in the recent era—A nationwide survey in Japan. J. Hepato-Biliary Pancreat. Sci..

[16] Amygdalos I., Müller-Franzes G., Bednarsch J., Czigany Z., Ulmer T.F., Bruners P., Kuhl C., Neumann U.P., Truhn D., Lang S.A. (2023). Novel machine learning algorithm can identify patients at risk of poor overall survival following curative resection for colorectal liver metastases. J. Hepato-Biliary Pancreat. Sci..

[17] Conticchio M., Uldry E., Hübner M., Digklia A., Fraga M., Sempoux C., Raisaro J.L., Fuks D. (2025). Prognostic Factors in Colorectal Liver Metastases: An Exhaustive Review of the Literature and Future Prospectives. Cancers.

[18] Li Y., Xu T., Wang X., Jia X., Ren M., Wang X. (2023). The prognostic utility of preoperative neutrophil-to-lymphocyte ratio (NLR) in patients with colorectal liver metastasis: A systematic review and meta-analysis. Cancer Cell Int..

[19] Margonis G.A., Vauthey J.N. (2022). Precision surgery for colorectal liver metastases: Current knowledge and future perspectives. Ann. Gastroenterol. Surg..

[20] Maki H., Jain A.J., Haddad A., Lendoire M., Chun Y.S., Vauthey J.N. (2023). Locoregional treatment for colorectal liver metastases aiming for precision medicine. Ann. Gastroenterol. Surg..

[21] Takematsu T., Mima K., Hayashi H., Kitano Y., Nakagawa S., Hiyoshi Y., Okabe H., Imai K., Miyamoto Y., Baba H. (2024). RAS mutation status in combination with the JSHBPS nomogram may be useful for preoperative identification of colorectal liver metastases with high risk of recurrence and mortality after hepatectomy. J. Hepato-Biliary Pancreat. Sci..

[22] Adam R., Haller D.G., Poston G., Raoul J.L., Spano J.P., Tabernero J., Van Custem E. (2010). Toward optimized front-line therapeutic strategies in patients with metastatic colorectal cancer–An expert review from the International Congress on Anti-Cancer Treatment (ICACT) 2009. Ann. Oncol..

[23] Noda T., Takahashi H., Tei M., Nishida N., Hata T., Takeda Y., Ohue M., Wada H., Mizushima T., Asaoka T. (2023). Clinical outcomes of neoadjuvant chemotherapy for resectable colorectal liver metastasis with intermediate risk of postoperative recurrence: A multi-institutional retrospective study. Ann. Gastroenterol. Surg..

[24] Nordlinger B., Sorbye H., Glimelius B., Poston G.J., Schlag P.M., Rougier P., Bechstein W.O., Primrose J.N., Walpole E.T., Finch-Jones M. (2008). Perioperative chemotherapy with FOLFOX4 and surgery versus surgery alone for resectable liver metastases from colorectal cancer (EORTC Intergroup trial 40983): A randomised controlled trial. Lancet.

[25] Nordlinger B., Sorbye H., Glimelius B., Poston G.J., Schlag P.M., Rougier P., Bechstein W.O., Primrose J.N., Walpole E.T., Finch-Jones M. (2013). Perioperative FOLFOX4 chemotherapy and surgery versus surgery alone for resectable liver metastases from colorectal cancer (EORTC 40983): Long-term results of a randomised, controlled, phase 3 trial. Lancet Oncol..

[26] Eisenhauer E.A., Therasse P., Bogaerts J., Schwartz L.H., Sargent D., Ford R., Dancey J., Arbuck S., Gwyther S., Mooney M. (2009). New response evaluation criteria in solid tumours: Revised RECIST guideline (version 1.1). Eur. J. Cancer.

[27] Igami T., Hayashi Y., Yokyama Y., Mori K., Ebata T. (2024). Development of real-time navigation system for laparoscopic hepatectomy using magnetic micro sensor. Minim. Invasive Ther. Allied Technol..

[28] Boretto L., Pelanis E., Regensburger A., Fretland Å.A., Edwin B., Elle O.J. (2024). Hybrid optical-vision tracking in laparoscopy: Accuracy of navigation and ultrasound reconstruction. Minim. Invasive Ther. Allied Technol..

[29] Grivennikov S.I., Greten F.R., Karin M. (2010). Immunity, inflammation, and cancer. Cell.

[30] Ichida H., Mise Y., Ito H., Ishizawa T., Inoue Y., Takahashi Y., Shinozaki E., Yamaguchi K., Saiura A. (2019). Optimal indication criteria for neoadjuvant chemotherapy in patients with resectable colorectal liver metastases. World J. Surg. Oncol..

[31] Loupakis F., Cremolini C., Masi G., Lonardi S., Zagonel V., Salvatore L., Cortesi E., Tomasello G., Ronzoni M., Spadi R. (2014). Initial therapy with FOLFOXIRI and bevacizumab for metastatic colorectal cancer. N. Engl. J. Med..

[32] Cremolini C., Loupakis F., Antoniotti C., Lupi C., Sensi E., Lonardi S., Mezi S., Tomasello G., Ronzoni M., Zaniboni A. (2015). FOLFOXIRI plus bevacizumab versus FOLFIRI plus bevacizumab as first-line treatment of patients with metastatic colorectal cancer: Updated overall survival and molecular subgroup analyses of the open-label, phase 3 TRIBE study. Lancet Oncol..

[33] Gruenberger T., Bridgewater J., Chau I., García Alfonso P., Rivoire M., Mudan S., Lasserre S., Hermann F., Waterkamp D., Adam R. (2015). Bevacizumab plus mFOLFOX-6 or FOLFOXIRI in patients with initially unresectable liver metastases from colorectal cancer: The OLIVIA multinational randomised phase II trial. Ann. Oncol..

[34] Shindoh J., Tzeng C.W., Aloia T.A., Curley S.A., Zimmitti G., Wei S.H., Huang S.Y., Mahvash A., Gupta S., Wallace M.J. (2013). Optimal future liver remnant in patients treated with extensive preoperative chemotherapy for colorectal liver metastases. Ann. Surg. Oncol..

[35] Kanesada K., Tsunedomi R., Hazama S., Ogihara H., Hamamoto Y., Shindo Y., Matsui H., Tokumitsu Y., Yoshida S., Iida M. (2023). Association between a single nucleotide polymorphism in the R3HCC1 gene and irinotecan toxicity. Cancer Med..

[36] Peng Z., Zhu Z.R., He C.Y., Huang H. (2025). A meta-analysis: Laparoscopic versus open liver resection for large hepatocellular carcinoma. Minim. Invasive Ther. Allied Technol..

