# Supplementary material for: Refining Outcomes in Technically Resectable Colorectal Liver Metastases: A Simplified Risk Model and the Role of Preoperative Chemotherapy
Source: Cancers (Basel). 2026 Jan 12;18(2):227. doi: 10.3390/cancers18020227 (PMC12838617; doi:10.3390/cancers18020227)
Supplement: Supplementary file 1 [file cancers-18-00227-s001.zip › cancers-4065520-supplementary.pdf]

Figure S1. Analysis of RFS after R0 resection and OS after liver resection of the patients with CRLM (n=115)

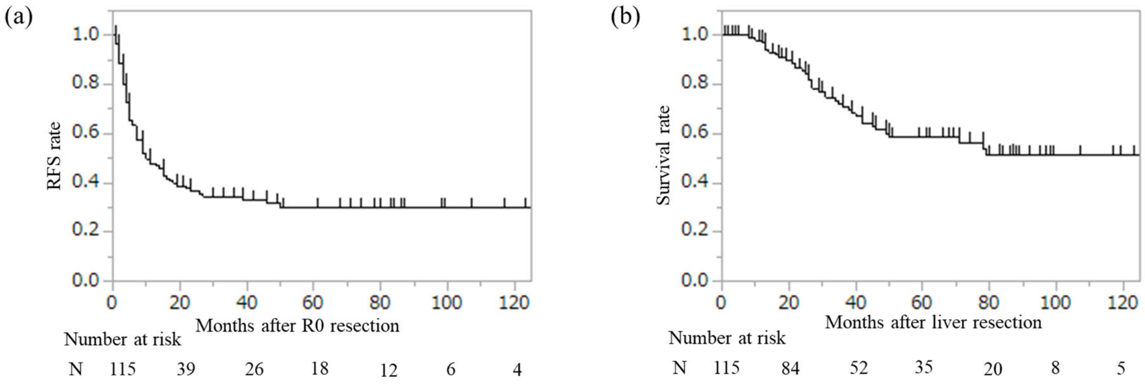

Table S1. Univariate and multivariate analysis of risk factors associated with recurrence after R0 resection for CRLM (n=115)

**Risk Factors**		***n* (%)**	**Univariate**				**Multivariate**			
			**HR**	**95%CI**		***p* value**	**HR**	**95%CI**		***p* value**
Age (years-old)	< 65	44 (38.3)	1	–	–	–				
	≥65	71 (61.7)	1.10	0.69	1.78	0.701				
Gender	Male	71 (61.7)	1	–	–	–				
	Female	44 (38.3)	1.07	0.66	1.71	0.768				
Primary site	Right	38 (33.0)	1	–	–	–				
	Left	77 (67.0)	0.90	0.56	1.48	0.679				
Primary tumor T status	pT1-3	91 (79.1)	1	–	–	–				
	pT4	23 (20.0)	0.98	0.55	1.68	0.954				
Primary tumor LN status	Negative	36 (31.3)	1	–	–	–				
	Positive	76 (66.1)	1.28	0.77	2.19	0.348				
ly (primary tumor)	Negative	13 (11.3)	1	–	–	–	1	–	–	–
	Positive	95 (82.6)	1.92	0.90	4.98	0.096	1.69	0.78	4.42	0.201
v (primary tumor)	Negative	27 (23.5)	1	–	–	–				
	Positive	82 (71.3)	0.89	0.53	1.59	0.683				
CEA level*	≤ 5.0 ng/ml	30 (26.1)	1	–	–	–	1	–	–	–
	> 5.0 ng/ml	85 (73.9)	1.83	1.06	3.36	0.029	1.26	0.68	2.45	0.466
CA19-9 level*	≤ 37.0 U/ml	76 (66.1)	1	–	–	–	1	–	–	–
	> 37.0 U/ml	39 (33.9)	1.66	1.03	2.66	0.039	1.03	0.59	1.78	0.919
Timing of diagnosis of CRLM**	Metachro-nous	20 (17.4)	1	–	–	–				
	Synchronous	95 (82.6)	1.67	0.90	4.37	0.110				

Extrahepatic metastatic disease*	No	101 (87.8)	1	–	–	–				
	Yes	14 (12.2)	1.29	0.62	2.40	0.472				
NLR***	< 2.9	89 (77.4)	1	–	–	–	1	–	–	–
	≥2.9	26 (22.6)	2.18	1.26	3.62	0.006	2.00	1.11	3.47	0.022
Prognostic criteria	low risk	69 (60.0)	1	–	–	–	1	–	–	–
	high risk	46 (40.0)	2.56	1.59	4.12	< 0.001	2.05	1.22	3.45	0.007

Univariate and multivariate analyses were analyzed using the Cox proportional hazards model. In the multivariate analysis, factors were extracted based on p values less than 0.10. *at diagnosis of CRLM, **Synchronous: Liver metastases are present at the time of diagnosis of the primary tumor, or liver metastases appear within 1 year after resection of the primary tumor. Metachronous: Liver metastases appear more than 1 year after resection of the primary tumor, ***before liver resection. ly: lymphatic vascular invasion, v: venous invasion, CEA: carcinoembryonic antigen, CA19-9: carbohydrate antigen 19-9, NLR: neutrophil-lymphocyte ratio.

Table S2. Univariate and multivariate analysis of risk factors associated with survival after liver resection for CRLM (n=115)

**Risk Factors**		***n* (%)**	**Univariate**				**Multivariate**			
			**HR**	**95%CI**		***p* value**	**HR**	**95%CI**		***p* value**
Age (years-old)	< 65	44 (38.3)	1	–	–	–				
	≥65	71 (61.7)	1.59	0.83	3.22	0.166				
Gender	Male	71 (61.7)	1	–	–	–				
	Female	44 (38.3)	1.25	0.65	2.38	0.494				
Primary site	Right	38 (33.0)	1	–	–	–				
	Left	77 (67.0)	1.46	0.73	3.15	0.294				
Primary tumor T status	pT1-3	91 (79.1)	1	–	–	–				
	pT4	23 (20.0)	0.60	0.23	1.35	0.232				
Primary tumor LN status	Negative	36 (31.3)	1	–	–	–				
	Positive	76 (66.1)	1.73	0.83	4.06	0.147				
ly (primary tumor)	Negative	13 (11.3)	1	–	–	–				
	Positive	95 (82.6)	1.37	0.55	4.59	0.535				
v (primary tumor)	Negative	27 (23.5)	1	–	–	–				
	Positive	82 (71.3)	0.73	0.37	1.54	0.397				
CEA level*	≤ 5.0 ng/ml	30 (26.1)	1	–	–	–	1	–	–	–
	> 5.0 ng/ml	85 (73.9)	2.98	1.27	8.72	0.010	2.20	0.90	6.60	0.086
CA19-9 level*	≤ 37.0 U/ml	76 (66.1)	1	–	–	–				
	> 37.0 U/ml	39 (33.9)	1.18	0.60	2.24	0.622				
Timing of diagnosis of CRLM**	Metachronous	20 (17.4)	1	–	–	–				

	Synchronous	95 (82.6)	1.40	0.60	4.10	0.462				
Extrahepatic metastatic disease*	No	101 (87.8)	1	–	–	–				
	Yes	14 (12.2)	1.14	0.39	2.68	0.786				
NLR***	< 2.9	89 (77.4)	1	–	–	–	1	–	–	–
	≥2.9	26 (22.6)	2.23	0.94	4.71	0.068	1.63	0.68	3.49	0.258
Prognostic criteria	low risk	69 (60.0)	1	–	–	–	1	–	–	–
	high risk	46 (40.0)	2.72	1.43	5.33	0.002	2.24	1.16	4.47	0.017

Univariate and multivariate analyses were analyzed using the Cox proportional hazards model. In the multivariate analysis, factors were extracted based on p values less than 0.10. *at diagnosis of CRLM, **Synchronous: Liver metastases are present at the time of diagnosis of the primary tumor, or liver metastases appear within 1 year after resection of the primary tumor. Metachronous: Liver metastases appear more than 1 year after resection of the primary tumor, ***before liver resection. ly: lymphatic vascular invasion, v: venous invasion, CEA: carcinoembryonic antigen, CA19-9: carbohydrate antigen 19-9, NLR: neutrophil-lymphocyte ratio.
